# Analysis of Spontaneously Reported Adverse Drug Events: Towards Developing Systems for Preventability

**DOI:** 10.1155/2024/1906797

**Published:** 2024-08-30

**Authors:** Courage Edem Ketor, Charles Kwaku Benneh, Emmanuel Sarkodie, Juliet Ama Anaglo, Adelaide Mensah, Samuel Owusu Somuah, Selorm Akakpo, Eric Woode

**Affiliations:** ^1^ Pharmacy Department Jasikan District Hospital Ghana Health Service, Jasikan, Ghana; ^2^ Department of Clinical Pharmacy and Pharmacy Practice School of Pharmacy and Pharmaceutical Sciences Ulster University, Coleraine, UK; ^3^ University Hospital Kwame Nkrumah University of Science and Technology, Kumasi, Ghana; ^4^ Peki Dzake Health Centre Ghana Health Service, Peki, Ghana; ^5^ Department of Pharmaceutics School of Pharmacy University of Health and Allied Sciences, Ho, Ghana; ^6^ Department of Pharmacy Practice School of Pharmacy University of Health and Allied Sciences, Ho, Ghana; ^7^ Pharmacy Department Ho Teaching Hospital, Ho, Ghana; ^8^ Department of Pharmacology and Toxicology School of Pharmacy University of Health and Allied Sciences, Ho, Ghana

**Keywords:** ADR, dechallenge, FDA, Ghana, preventability

## Abstract

**Background:** Analysing data on adverse drug reactions (ADRs) in health facilities is an essential step to help develop effective strategies to reduce their incidence. The objective was to analyse spontaneous ADR reports sent to the Ghanaian Food and Drugs Authority (FDA) by two reporting health facilities over 5 years.

**Methods:** Data from duplicate spontaneous ADR reports sent to the FDA (Ghana) from 2014 to 2018 were extracted. The relationship between independent variables such as age, sex, and source of drugs and ADR outcomes was assessed with either chi-square or a Cramer's V test for association where appropriate.

**Results:** Type A reactions (65.2%) were the most prevalent of the ADRs, followed by Type B (34.1%), with the majority (80%) of patients affected recovering fully. The majority of Type A reactions (54.1%) occurred in the clinic, while the majority of Type B reactions (43.5%) occurred in the hospital. The skin and central nervous system (CNS) were the most affected (70.8%) organs. A higher incidence of CNS and skin-related ADRs was recorded in patients older than 30 (RR = 1.28 (1.07–1.53)). Also, females were more likely to experience a CNS-related ADR. The seriousness of the ADR was found to be significantly associated with the (1) type of prescriber, (2) whether the drug was prescribed, or (3) whether the drug regimen prescribed was appropriate. Even though, in 86% of cases, the offending drug was withdrawn within the first 5 days, it exceeded 20 days in about 6% of cases. The record of allergy status in a patient's folder and the source of the drug were significantly associated with the chance that the offending drug was withdrawn. However, recording ADRs did not influence whether the offending drug was stopped.

**Conclusion:** Most of the ADRs experienced by patients could be avoided if the current systems are improved to prevent the rechallenge of offending drugs. Efforts to improve and update patient medication records and steps to ensure continuity of care are essential in preventing these adverse drug events.

## 1. Introduction

Medicines have become one of the most crucial components of healthcare systems. When administered, they aim to reach a set efficacy, but in some cases, the drug exposure leads to unintended effects and sometimes adverse reactions. Adverse drug reactions (ADRs) are “any noxious, unintended, undesired effect of a drug that occurs at the dosage used in humans for prophylaxis, diagnosis, and therapy” [1]. According to the European Medicines Agency and Heads of Medicines Agencies [2], adverse events arise from the use of medicinal products within or outside marketing authorizations or as a result of occupational exposures. Therefore, unintended or noxious experiences from using drugs outside marketing authorization in situations of abuse, off-label use, misuse, overdose, and medication errors are also referred to as adverse events [2]. The adverse reactions of drugs can range from relatively mild to, in rare cases, severe and life-threatening.

In many low- and middle-income countries (LMIC), there is a lack of scientific evidence on the local burden of harm caused by drugs and how they could be prevented [3]. There is enough evidence worldwide that ADRs directly impact the cost of care and disease management [4–6]. Therefore, strategies to prevent the occurrence and reduce the impact of ADRs are necessary to improve patient safety. About a third of ADRs are preventable, and the impact of most ADRs is significantly reduced when the offending drug is withdrawn from the patient's drug regimen [7].

In Ghana, reporting spontaneous ADRs is the most widely used and cost-effective method for monitoring the safety of approved drugs. However, this method is often associated with a high degree of underreporting by healthcare professionals [8, 9]. Irrespective of the low reporting levels, determining the prevalence of ADRs has been demonstrated to be a useful indicator for improving healthcare delivery. Every healthcare system's persistent objective should include decreasing ADRs by implementing suitable prevention strategies. It is, therefore, pivotal not only to increase the education of patients and prescribers on spontaneous ADR reporting and documentation but also to analyse individual facility records to determine trends and, if possible, propose evidence-based strategies to avoid or prevent ADRs, improving patient safety.

This body of work analysed spontaneous reports sent to the Ghanaian Food and Drugs Authority (FDA) from two reporting health facilities over 5 years. The objective was to identify and classify the ADRs, identify the drugs commonly implicated in the ADR, and propose preventability measures to reduce the incidence and avoid rechallenge. We further assessed the distribution and association between patient characteristics and ADR outcomes.

## 2. Methods

### 2.1. Study Sites

Two health facilities, the Jasikan District Hospital (JDH) and Peki Dzake Health Centre (PDHC), with the highest reporting of ADRs in the Volta regions of Ghana, were selected. JDH and PDHC have a daily average attendance of 165 and 70 patients, respectively.

### 2.2. Study Design and Data Collection

This was a retrospective cross-sectional design. Using a data collection tool, the total sampling technique was applied to select all eligible cases from anonymised patient ADR records (see Supporting Information S2). The study population consisted of all available reported cases over a 5-year period (i.e., 145). Duplicate spontaneous reports to the Ghanaian FDA were reviewed over 5 years (2014–2018). Deidentified patients' clinical records were also reviewed for the outcome of care, grade of professional making diagnosis, management of ADR, and possibilities of rechallenge.

### 2.3. Ethics and Patient Consent

Ethical approval for this study was waived by the institutional review boards of the JDH and PDHC because the facility provided anonymised patient ADR records and deidentified patient records from which we extracted the data for this study. Informed consent was not sought for the present study because no study participants were contacted to provide information about their ADR. Our study relied solely on data from the records of the patients who met the inclusion criteria from 2014 to 2018.

### 2.4. Data Analysis

All ADRs were categorized by drug, drug class, severity, probability of causality as classified by the Ghanaian FDA-Technical Advisory Committee, demographics, and treatment outcome.

The general classification of the medicine by the Ghanaian FDA according to the National Essential Medicine List and the Ghanaian Standard Treatment Guidelines was used to classify the medicines implicated. The classification of the ADR was also done according to the recommendation of the Ghanaian Food and Drug Authority.

Frequency tables were used to summarise frequency data from the demographic characteristics of participants and a summary of ADR characteristics.

We investigated the association of nine independent variables (Table 1) with 10 outcome variables (Table 2). Association between categorical variables was assessed using either a chi-square test for association or Cramer's V test. Chi-square analysis was conducted to determine the relationship between two dichotomous variables. Cramer's V test was used to provide information about the strength and significance of the association between the dependent and independent variables when the assumptions and conditions for other association tests, such as chi-square and Fisher's exact test, were not met.

Statistical analyses were performed using the SPSS Statistics 25 (IBM, USA) and SigmaPlot® version 12.5 for Windows (Systat Software Inc., USA). In all the analyses, a *p* value less than 0.05 was considered significant.

## 3. Results

### 3.1. Patient and Medicine-Related Characteristics

**Figure 1:**
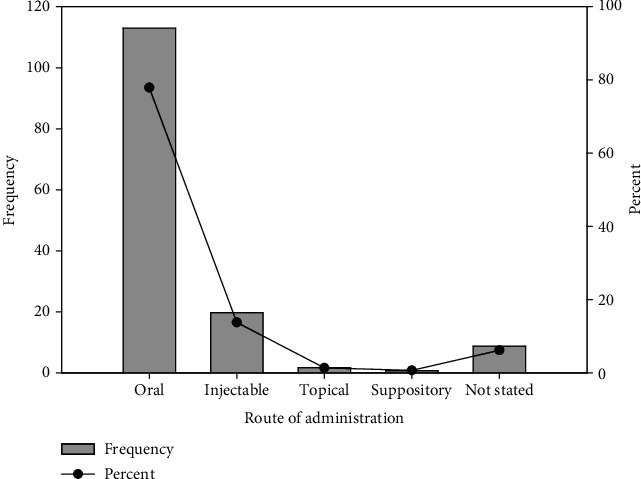
Route of administration associated with the adverse drug reactions (ADRs) observed.

**Figure 2:**
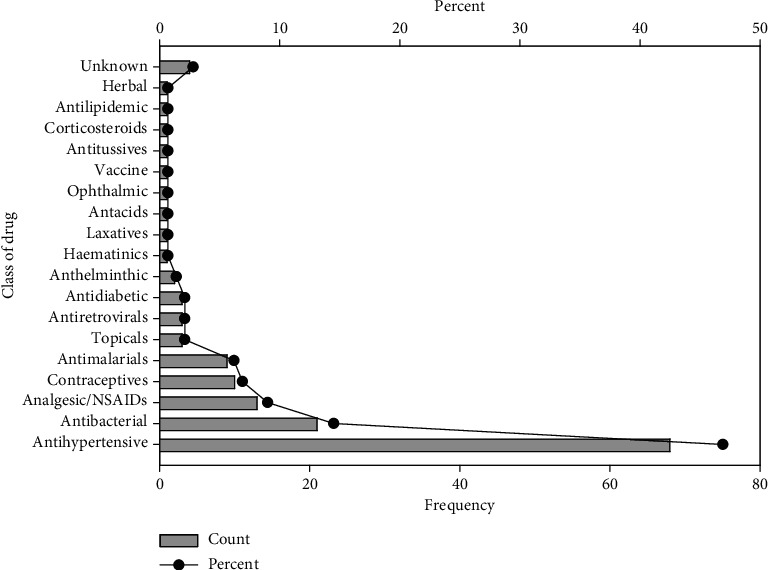
Data showing the frequency (bar graph) and percentage (line graph) contribution of the classes of drugs to the adverse drug reactions (ADRs) reported.

**Figure 3:**
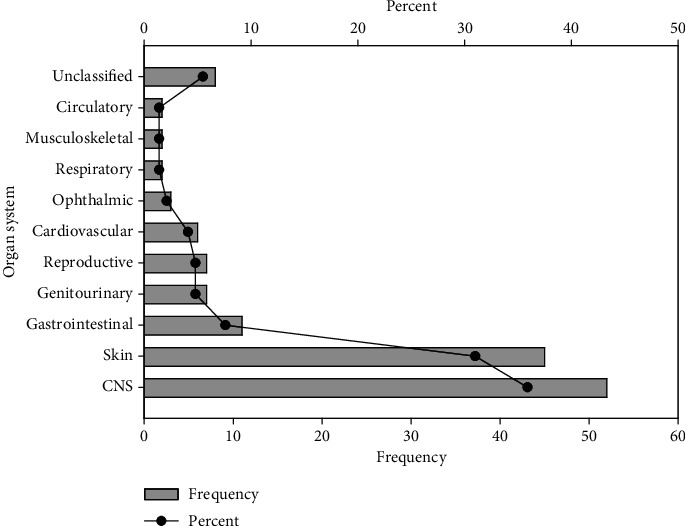
Distribution of the organ system implicated in the ADRs.

Females recorded the highest number of ADRs of 99 (73.9%). The mean age and weight of the population reviewed were 52.6 (*SD* = 21.7) and 64.5 (*SD* = 48.9), respectively (Table 3). Most ADRs (*n* = 113, 77.9%) resulted from oral medications (Figure 1). Antihypertensives (*n* = 68, 46.9%), antibacterial (*n* = 21, 14.5%), analgesics/anti-inflammatory agents (*n* = 13, 9%), contraceptives (*n* = 10, 6.9%), and antimalarials (*n* = 9, 6.2%) were found to be classes of medication causing ADRs in the two facilities (Figure 2). The central nervous system (CNS) was the organ system most affected, with a percentage of 35.9% (*n* = 52), followed by the skin and gastrointestinal systems, with percentages of 31% (*n* = 45) and 7.6% (*n* = 11), respectively (Figure 3).

### 3.2. Characteristics of ADRs

**Figure 4:**
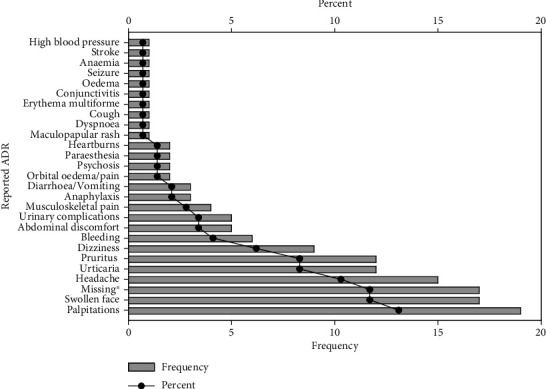
Prevalence of the reported ADRs in the two facilities.

The common presentations of ADR within the population studied include palpitations (*n* = 19, 13.1%), swollen lips, face and mouth (*n* = 17, 11.7%), headache (*n* = 15, 10.3%), urticarial skin rash (*n* = 12, 8.3%), heart burns (*n* = 9, 6.2%), pruritus (*n* = 9, 6.2%) and abdominal discomfort (*n* = 6, 4.1%) (Figure 4).  Table 4 summarizes all complaints and adverse reactions described in this study. The most common type of ADR observed was type A (*n* = 86, 65.2%), followed by type B (*n* = 45, 34.1%). While most patients recovered from ADRs (*n* = 125, 92.6%), one died, with 22 hospitalized. Even though the majority of causality feedback was yet to be received, out of 13 that were received, nine ADRs were adjudged to be possible, three certain, and one probable (Table 4).

Antihypertensive were the most common medications implicated in the ADRs experienced by patients. Amlodipine, lisinopril/hydrochlorothiazide, and nifedipine were implicated in most of the adverse effects registered. Selected antibiotics, mainly amoxicillin, amoxicillin/clavulanic acid, ciprofloxacin and the antimalarial, and artemether/lumefantrine, were linked to ADRs in about 4% of the patients. Among the analgesics, tramadol was implicated in about 5% of patients' ADRs, with diclofenac, ibuprofen, and paracetamol recorded to be responsible for relatively fewer ADRs among the population in this study.

**Figure 5:**
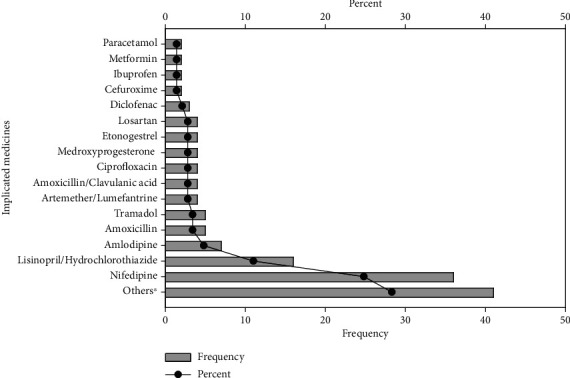
Distribution of medications causing ADRs in the two facilities.

**Figure 6:**
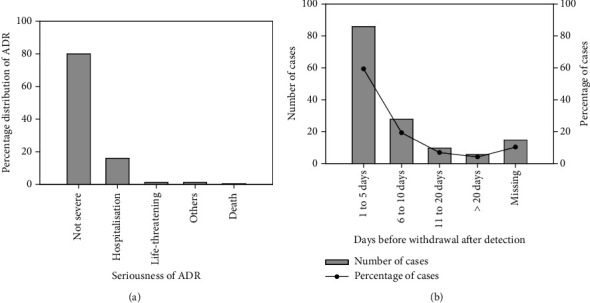
Frequency distribution of the (a) percentage distribution of the seriousness of ADRs and the (b) duration of exposure (days) to offending medication before withdrawal after detection.

Unfortunately, in about 8.3% of ADR reports, the suspected or implicated medication was not indicated (Figure 5). Most ADRs (i.e., 80.2% of 145 sampled) did not lead to fatal outcomes. About 16% (16.2%) of patients who experienced ADRs were hospitalized, with most patients recognizing the untoward reactions to medications after just a day on the offending drug (*n* = 37, 25.5%) (Figure 6(a)). It is assumed that the severity of the ADR was an influence on hospitalization. The specific reason aside the severity of ADR is not captured in our data collection tool. However, in clinical practice, hospitalized cases cover those that cannot be managed in the outpatient department. When adverse reactions were reported, the (suspected) offending drug was withdrawn within 5 days in 86% of cases, while the time to withdrawal exceeded 20 days in 6% of cases (Figure 6(b)).

### 3.3. Association Between Age or Gender and ADR Outcome Variables

**Figure 7:**
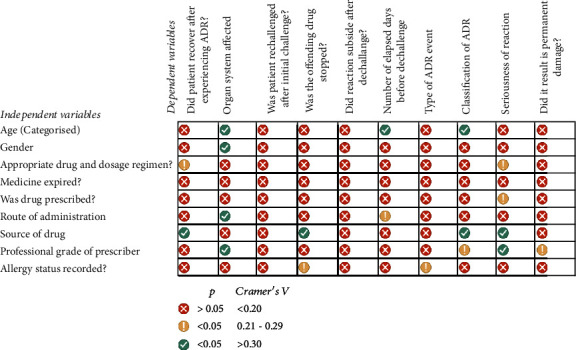
A matrix grid summarising the association or lack thereof between the selected dependent and independent variables. Cramer's V test revealed an association between selected independent variables (rows) and dependent variables (columns).

Cramer's V statistic was conducted to identify the possible association between age and selected dependent variables. We identified a significant association between the age categories and organ system affected by the ADR, type of ADR, and the number of days elapsed before dechallenge was initiated (Table 5 and Figure 7). The skin and CNS were the most affected (70.8%), while the respiratory system and the eye, on the other hand, were the least (0.04%) associated with ADRs. In addition to the above, a higher incidence of CNS and skin-related ADRs were recorded with increasing age categories. A Pearson chi-square test indicated that patients who are <30 years and patients older than 30 years were significantly different on whether or not they will experience a CNS or skin-related ADR (*χ*^2^ = 8.89, *df* = 1, *N* = 95, *p* = .003, phi = .306). Patients older than 30 years are more likely (risk ratio = 1.28 (1.07–1.53)) than expected to have a higher incidence of CNS-related ADR compared to patients < 30 years old. Examination of the standardized residuals indicates that the proportion of patients 30 years or younger contributed to the significant results. In terms of the type of ADR, a dose-related (type A) ADR was experienced by about two-thirds of patients (65.2%), while about a third (34.1%) experienced a nondose-related ADR (type B). A negligible proportion of patients in this study experienced a type F ADR. An increase in age was associated with a higher proportion of dose-related ADR, with the proportion of the incidence of types A or B being highest among patients over 60 years. Within all age categories, the offending drug was withdrawn within a 7-day for most patients (80.8%). A decreasingly smaller proportion of patients had withdrawal between 8 and 14 days (7.69%), 15–21 days (4.62%), and beyond 22 days (3.85%).

As mentioned earlier, the incidence of skin- and CNS-related ADRs formed most reported cases, with varied proportions of males and females who experienced these ADRs (Table 6). Using a Pearson chi-square test, we identified that female patients compared to male patients were significantly more likely to experience a CNS compared to skin-related ADR (*χ*^2^ = 4.20, df = 1, *N* = 95, *p* = 0.04, phi = 0.210) (Table 6).

### 3.4. Association Between Drug-Related Variables and ADR Outcome Variables

The seriousness of the adverse drug event was moderately associated with the appropriateness of the drug regimen and whether the drug was prescribed (Tables 7 and 8). In most cases (86.6%), a medical professional prescribed the dosage regimen associated with the adverse drug event. The majority (94.1%) of the dosage regimen linked to the adverse drug event was found to be appropriate after further review of patient records. Further, the seriousness of the ADR had no association with whether the offending medication was prescribed or otherwise (Table 9).

The route of administration was strongly correlated with the organ system affected but weakly correlated with the number of days before the dechallenge was initiated (Table 8). All offending topical, most oral (80.3%) and some injectable (66.7%) formulations were withdrawn within the first 7 days of the adverse drug event being reported. However, a smaller proportion of patients who experienced ADRs after taking orally administered drugs still received the offending drug for a period of 8–14 days (10.7%), 15–21 days (4.5%), and more than 3 weeks (4.5%) before dechallenge was initiated (Table 8).

Interestingly, the type of ADR a patient experiences, whether the use of the offending drug is discontinued, and the seriousness and prognosis of the ADR have all been found to be strongly correlated with the drug source. Upon closer examination, it was discovered that the majority of type A reactions (54.1%) occur in clinics, while the majority of type B reactions (43.5%) occur in hospitals (Table 10). One surprising finding was that, even after registering an adverse drug event, the drugs supplied by community pharmacies were less likely to be stopped in 42% of cases. In terms of recovery from the ADRs, we identified that the pattern of recovery parallels the trend with which the offending drug is identified and removed from the patient's treatment regimen (Table 10).

### 3.5. Association Between Prescriber Level or ADR Records and ADR Outcome Variables

We further evaluated the relationship between the healthcare provider making the diagnosis and the dependent variables, such as but not limited to the seriousness of the ADR, the classification of the ADR, whether the ADR led to an unfavourable medical condition, and the organ system involved in the ADR (Tables 11 and 12). We found a strong correlation between the aforementioned. Less than 10% (8.89%) of ADRs had adverse effects that necessitated immediate medical attention. More than 80% of ADR experienced by patients were classified as not serious, with only about 16% of ADR requiring hospitalization. Only 0.7% and 1.4% of patients who experienced ADR passed away or experienced a life-threatening event, respectively (Table 11).

A higher proportion of types A and B reactions were more associated with physician assistants (94.2% and 66.7%, respectively) than doctors (5.8% and 28.8%, respectively). There was a significant association between the medical professional making the diagnosis and prescribing and the organ system the ADR affects if it occurs (Table 12). A closer look at the data revealed that there were reduced odds (0.047 (CI = 0.006–0.379)) of a patient experiencing a CNS-related ADR when a doctor makes the diagnosis and prescribes the drug as compared to physician assistants (*χ*^2^ = 14.41, df = 1, *N* = 93, *p* < 0.001, phi = −0.394).

Less than half (44.4% (*n* = 59)) of ADRs were not recorded in patient folders, and suspected offending drugs were not stopped in about 3.4% (*n* = 2) of cases, as opposed to 33.8% (*n* = 45) of ADRs recorded, with about 12.5% (*n* = 6) suspected offending drugs not being stopped. Whether the record of ADR in a patient's medical information was related to the type of ADR a patient experiences or whether it informed the removal of the offending drug from the patient's management plan, we found a weak but significant association (Table 12).

## 4. Discussion

Adverse drug events are untoward and undesired effects that may occur, in most cases, due to the extension of inherent pharmacodynamic properties or interaction with administered drugs. However, a minority of such reactions are idiosyncratic. The implications of these medicine-induced reactions impact disease prognosis and quality of life and, in a few instances, can lead to mortality irrespective of age or gender.

Even though it is currently known that the cause of ADR is multifactorial, efforts to identify drugs commonly associated with ADR have helped in targeted educational interventions first to identify the occurrence of the ADR, implement mitigating measures, and subsequently report to the appropriate stakeholders. In Ghana, such reports are made to the Ghanaian FDA using a standard ADR reporting form (blue form), which requires entries of patient details, details of adverse reactions and treatment given, outcome of adverse reactions, suspected product(s), and any concomitant drugs the patient was on at the time of the reaction. Due to the limited information in these forms, additional patient information was gathered from patient records after appropriately deidentifying patient's unique details.

While the influence of age on the pharmacokinetics and pharmacodynamic effects of drugs is well known and factored into prescribing, the influence of sex, although well understood, for some drugs is often considered impractical to implement in routine care. It is, therefore, not surprising that females are more likely to experience a greater risk of experiencing a medicine-related adverse effect [10–14]. Although the reason(s) for the higher risk in females is unclear, sex-related differences in pharmacokinetic, immunological and hormonal factors, and differences in medication utilization patterns by women compared with men could account for the increased risk in females [15]. Also, some studies have identified that females are more likely than men to report nonserious ADR to healthcare professionals [16–18].

In this study, females (*n* = 99, 73.9%) formed a greater proportion of patients who experienced and reported ADRs. We further identified that female patients were more likely to experience CNS adverse effects compared to males. This disproportionate difference in sex difference has roots in the unbalanced design and implementation of most clinical studies [19]. The incidence of ADRs was fairly distributed among the age groups. As expected, there was a strong association between older patients and the incidence of developing a CNS-related ADR [20]. We also identified that advanced age was associated with a higher proportion of dose-related ADRs, with type A being highest among patients over 60 years. A good knowledge of drug pharmacology and an increased emphasis on dose individualisation in the older population will be integral to reducing the incidence of type A adverse reactions, which are predictable and mostly preventable [21, 22]. Chronic (type C) and delayed (type D) adverse reactions were not observed in this study, possibly because this study is based on retrospective data that records details at the specific time the ADR occurs. More than 90% of reactions abated after withdrawal of the suspected drug, which confirms the findings of Mayathevar and colleagues, who measured similar recovery rates [23]. While a greater proportion of patients did not develop any untoward medical condition as a consequence of the ADR, a small proportion were hospitalized, out of which developed life-threatening conditions with one case leading to death. The latter was due to a phenoxymethylpenicillin-induced anaphylaxis.

With the mean age of patients in this study being more than 50 years, it was unsurprising that a greater proportion of medication used was to manage cardiovascular conditions [24, 25]. Nifedipine, a dihydropyridine calcium channel blocker (CCB), recorded the highest incidence of ADRs among the antihypertensives, followed by lisinopril/hydrochlorothiazide. However, the ACE-I component of the latter was the suspected medication reported to the FDA (Ghana) based on the pharmacology of the drug and the predictability of its effects [26]. These observations are consistent with findings from studies conducted to monitor ADRs caused by antihypertensives, which found that CCBs were implicated in most ADRs, followed by beta-blockers, angiotensin-converting enzyme inhibitors and diuretics [27, 28].

With hypertension being the highest cause of morbidity burden, only second to malaria, at the two healthcare facilities, it is not out of kilter that most drugs investigated to have ADRs where antihypertensives, although these drugs can also be used to manage other related cardiovascular and renal conditions. When collecting this data, the two facilities managed hypertension according to the Joint National Committee 8 guidelines for managing hypertension in adults, which recommends using CCBs as one of the first-line drug options in the black population. The most common presenting complaints were palpitations, headache, angioedema, skin rash or urticaria rash, heartburns, and abdominal discomforts. Angioedema frequently presented as facial, lips, and tongue swelling, confirming earlier descriptions by Saltoun and Metzger [29] and Morimoto et al. [30]. Although measurement of the association between drug use and patient harm by using cause-effect diagnosis is complex, and coupled with the fact that most events are nonspecific and likely to be confused with clinical manifestations of current disease conditions, the Technical Advisory Committee of the FDA communicates causality to reporting facilities. Out of about 136 reports, only 15 causality feedbacks were available for analysis [31]. Nine of the 15 feedbacks received were classified as “possible,” while 4 and 2 reports were classified as “certain” and ‘”probable” according to the WHO-UMC Scale. The remaining were categorized as “yet to be received” at the facility for this study.

A justifiable case for the need for preventability measures was observed in many instances. In some instances, patients who experienced angioedema while on prescribed oral lisinopril/hydrochlorothiazide were prescribed the same drug at least a month after the initial challenge. Two main reasons are likely to be responsible for these lapses above and additional medical errors that lead to preventable ADRs (pADRs).

First, the lack of coordinated health management systems could be responsible for some avoidable ADRs in this study. To target preventability, the hospital/clinical management software and patient records should be standardized to facilitate communication between healthcare facilities. Currently, there is little or no interoperability between hospital management systems, even for hospitals within the same region. From our experience, this shortfall results in duplication of medical records across health facilities and adversely impacts patients' continuity of care, especially those with chronic conditions. This negatively impacts the possibility of identifying patients with a specific ADR predisposition and increases the possibility of rechallenging an offending drug. There have been situations of rechallenge of offending medications in patients due to a lack of information on previous exposure. This mostly happens when patients attend a new facility. Interoperability of hospital management systems might avert this risk, especially systems with visible alert functions on patient allergies and previous serious ADRs.

This could also be attributed partly to overreliance on memory and documentation in clinical folders, which can fail when providers unintentionally forget or misplace clinical records.

In Ghana, like most parts of the world, the community pharmacy plays an important role in managing minor ailments, offering prescription refills and continuity of care for most patients. This study identified that drugs sourced from community pharmacies were less likely to be stopped after a patient experienced an ADR. This reveals the lack of continuity of care in the community pharmacy setting for patients prescribed or receiving drugs from the community pharmacies. The nonexistence of this relationship leads to delays in offering pharmaceutical care to patients and is partly responsible for some of the avoidable ADR patients experience. Community pharmacists' role can be leveraged in other jurisdictions to improve ADR reporting after significant education [32–34]. Considering the scope of knowledge and experience in medicine optimisation, community pharmacists will play an integral role in strategies to reduce the incidence of ADRs.

At the hospital level, there is a need for a local-level review of ADRs led by a functional drug and therapeutic committee (DTC) with the support of institutional contact persons (ICPs). The allergy card and the interoperability of health record systems should be considered as part of preventive strategies, as they have a greater chance of detecting previous exposures. With the support of ICPs, DTCs should be encouraged and trained to set up pharmacovigilance subcommittees to lead safety activities in health facilities.

We established that the indication of a patient's allergy or ADR status is associated with the type of ADR and the probability that an offending drug was withdrawn and not introduced as part of a future drug therapy regimen. In addition to the obvious, one possible solution is to empower patients to take particular interest in their medications and be provided with an alert system (ADR card) to remind healthcare providers of their peculiar drug response anywhere they seek care.

Patients can be empowered through education to take a special interest in their health. The health system can be equipped with an alert system to remind healthcare providers of patients' allergies anywhere they seek care. The ADR card, proposed to be an alert system to empower the patient, will be kept by the patient and shown to any healthcare provider anywhere they seek care, just like they do for hospital identification cards and Insurance Identity Cards.

Alert systems should be activated in the facility's electronic health management systems. Also, collaborative strategies should be developed to ensure the interoperability of electronic health management systems of health facilities to ensure e-tracking and prevent avoidable ADRs.

Future studies should consider investigating pADRs using preventability criteria using the Schumock and Thornton algorithm with modifications if necessary [35].

### 4.1. Limitation

The study design employed was a retrospective cross-sectional which described signals of reported ADRs to the Ghanaian FDA over a period in a snapshot without immediate cause-and-effect analysis for remedy. However, it helps to prevent future occurrences if care providers document, disseminate, and utilise signals well.

## 5. Conclusion

The adverse drug events experienced by patients could be avoided if the current systems are improved to prevent the rechallenge of patients with drugs established to induce ADRs. Strategies to improve and update patient medication records and allow continuity of care are essential in preventing these adverse drug events. An ADR card is proposed as an alert system to empower the patient and minimize the repetition of ADRs.

### 5.1. Recommendation

The current study's design limits the ability to pick up and assess ADRs with a delayed onset. In addition, there was limited data on paediatric ADRs. An analysis of an extensive database could reveal relevant paediatric cases. However, we recommend updating the standard ADR reporting form (blue form) to include a medical history, past drug history and allergy status (e.g., G6PD deficiency) to serve as a composite form for analysis since folders do not accompany reports during submission. A proposed risk minimization strategy in the form of a designed allergy card with the minimum information that could help prevent the rechallenge of patients to a suspected/implicated drug in the future (see below).

## Figures and Tables

**Table 1:** Details of the independent variable categories used for further analysis.

**Classification**	**Independent variable**
Demographics	a. Age (categorized)
	b. Gender

Medicine related	c. Appropriate drug and dosage regimen?
	d. Medicine expired?
	e. Was the drug prescribed?
	f. Route of administration
	g. Source of drug

Prescriber related	h. Professional grade of prescriber

Records/reporting related event	i. Is allergy status recorded?

**Table 2:** Details of dependent variable categories used for further analysis.

**Classification**	**Dependent variable**
Recovery from ADR	a. Did the patient recover after experiencing ADR?
	b. Organ system affected

Drug dechallenge related	c. Was the patient rechallenged after the initial challenge?
	d. Was the offending drug stopped?
	e. Did the reaction subside after dechallenge?
	f. Number of elapsed days before dechallenge

Adverse drug reaction	g. Type of ADR event
	h. Classification of ADR
	i. Seriousness of reaction
	j. Did it result in permanent damage?

**Table 3:** Characteristics of the participants sampled who experienced ADR.

**Variable**	**Levels**	**Frequency**	**%**	**Mean**	**SD**
Gender	Male	35	25.5		
	Female	99	72.3		
	Missing	3	2.2		

Age group	0–12	5	3.6	52.6	21.7
	13–18	3	2.2		
	19–45	42	30.7		
	46–64	43	31.4		
	65 and above	42	30.7		
	Missing	2	1.5		

Weight (kg)				64.5	48.9

Type of report	Patient related	137	94.5		
	Product related	8	5.5		

**Table 4:** Summary of characteristics of ADRs reported.

**Variable**	**Frequency**	**%**
*ADR type*		
Type A: dose related	86	62.8
Type B: nondose related	45	32.8
Type F: failure of therapy	1	0.7
Missing	5	3.6
*Event type (product related)*		
Therapeutic failures	7	87.5
Questionable components	1	12.5
*Recovered*		
Recovered	125	92.6
Not yet recovered	6	4.4
Unknown	6	3.0
*Untoward medical condition*		
No	123	89.8
Yes	12	8.8
Not indicated	2	1.4
*Seriousness of reaction*		
None	109	79.6
Hospitalization	22	16.0
Others	3	2.2
Life threatening	2	1.5
Death	1	0.7
*Source of ADR*		
Outpatient department	112	81.8
General ward	20	14.6
Antiretroviral centre	4	2.9
Maternity	1	0.7
*Causality of ADR*		
Certain	4	2.9
Probable/likely	2	1.5
Possible	9	6.6
Feedback yet to be received	122	89.1
*Professional making diagnosis*		
Physician assistant	117	85.4
Doctor	18	13.1
Nurse	2	1.5

**Table 5:** Relationship between the age of patients and the nature and characteristics of the ADR.

	**Age**						**V**	**p**
	**n**	**0–15**	**16–30**	**31–45**	**46–60**	**>60**		
*Organ system involved*							0.301	0.003
Skin	45	4	7	9	10	14		
CNS	52	1	1	10	13	26		
Eye	3	0	1	0	1	1		
Respiratory	2	1	0	1	0	0		
GIT	11	1	0	1	0	0		
CVS	6	0	0	2	1	3		
Genitourinary	7	0	0	1	3	3		
Other	11	0	6	5	0	0		
*Classification of ADR*							0.372	<0.001
Type A: dose related	86	4	5	18	26	33		
Type B: nondose related	45	1	10	7	6	19		
Type F: failure of therapy	1	1	0	0	0	0		
*Days until dechallange*							0.365	<0.001
0–7	105	5	13	18	26	42		
8–14	14	0	1	1	5	7		
15–21	6	1	1	2	0	2		
22–28	2	0	0	0	1	1		
>29	3	0	1	1	1	0		

Abbreviations: CNS = central nervous system; CVS = cardiovascular system; GIT = gastrointestinal tract.

**Table 6:** Relationship between the gender of the patient and the organ system affected.

	**Gender**			**V**	**p**
	**n**	**Male**	**Female**		
*Organ system involved*				0.352	0.020
Skin	44	17	27		
CNS	51	10	41		
Eye	3	0	3		
Respiratory	2	2	0		
GIT	11	2	9		
CVS	6	1	5		
Genitourinary	7	3	4		
Other	10	0	10		

Abbreviations: CNS = central nervous system; CVS = cardiovascular system; GIT = gastrointestinal tract.

**Table 7:** Association between the appropriateness of the dosage regimen and the seriousness of or recovery from the ADR.

	**Was the drug and dose appropriate?**			**V**	**p**
	**n**	**Yes**	**No**		
*Seriousness of reaction*				0.292	0.004
Death	1	1	0		
Life threatening	2	2	0		
Hospitalization	21	20	1		
None	107	104	3		
Others	2	2	0		
*Recovery*				0.212	0.019
Recovered	123	120	3		
Not recovered	6	6	0		
Unknown	3	2	1		

**Table 8:** Association between the route of administration and the organ system affected or days until the discontinuation of the offending drug.

	**n**	**Route of administration**				**V**	**p**
		**Oral**	**Topical**	**Injectable**	**Other**		
*Organ system involved*						0.402	<0.001
Skin	45	35	1	9	0		
CNS	52	51	0	1	0		
Eye	3	2	0	1	0		
Respiratory	2	2	0	0	0		
GIT	11	9	0	1	1		
CVS	6	6	0	0	0		
Genitourinary	7	6	1	0	0		
Other	10	2	0	8	0		
*Days until dechallange*						0.259	0.025
0–7	104	90	2	12	0		
8–14	14	12	0	1	1		
15–21	6	5	0	1	0		
22–28	2	2	0	0	0		
>29	7	3	0	4	0		

Abbreviations: CNS = central nervous system; CVS = cardiovascular system; GIT = gastrointestinal tract.

**Table 9:** Association between whether the offending drug was prescribed and the seriousness of the ADR.

	**Was the drug prescribed?**			**V**	**p**
	**n**	**Yes**	**No**		
*Seriousness of reaction*				0.281	0.268
Death	1	0	1		
Life threatening	2	2	0		
Hospitalization	22	21	1		
None	107	92	15		
Others	2	1	1		

**Table 10:** Association between the source of drug and the characteristics of and recovery from the ADR.

	**n**	**Source of drug**						**V**	**p**
		**Hospital**	**Community pharmacy**	**Chemical shop**	**Drug peddler**	**Could not ascertain**	**Clinic pharmacy**		
*Seriousness of reaction*								0.303	0.002
Death	1	0	0	1	0	0	0		
Life threatening	2	2	0	0	0	0	0		
Hospitalization	21	18	2	1	0	0	0		
None	109	35	1	8	4	5	47		
Others	2	0	1	1	0	0	0		
*Classification of ADR*								0.458	<0.001
Type A: dose related	85	18	8	8	2	3	46		
Type B: nondose related	45	37	1	3	2	2	0		
Type F: failure of therapy	1	0	0	0	0	0	1		
*Was the offending drug stopped?*								0.481	<0.001
Yes	126	54	7	10	4	5	46		
No	8	1	5	1	0	0	1		
*Recovery*								0.365	<0.001
Recovered	124	54	8	10	2	5	45		
Not recovered	6	1	3	1	1	0	0		
Unknown	1	1	0	0	1	0	0		

**Table 11:** Association between the professional making diagnosis and the characteristics of the ADR.

	**n**	**Professional making diagnosis**				
		**Doctor**	**Physician assistant**	**Nurse**	**V**	**p**
*ADR result in untoward medical condition*					0.262	0.010
Yes	12	4	7	1		
No	123	14	108	1		
*Seriousness of reaction*					0.335	>0.001
Death	1	1	0	0		
Life threatening	2	1	1	0		
Hospitalization	22	9	12	1		
None	109	7	101	1		
Others	2	0	2	0		
*Classification of ADR*					0.263	0.001
Type A: dose related	86	5	81	0		
Type B: nondose related	45	13	30	2		
Type F: Failure of therapy	1	0	1	0		
*Organ system involved*					0.319	0.015
Skin	45	13	31	1		
CNS	52	1	51	0		
Eye	3	1	2	0		
Respiratory	2	0	2	0		
GIT	11	1	10	0		
CVS	6	2	4	0		
Genitourinary	7	0	7	0		
Other	11	1	10	1		

Abbreviations: CNS = central nervous system; CVS = cardiovascular system; GIT = gastrointestinal tract.

**Table 12:** Association between the record of a patient's allergy status in the treatment folder and the ADR characteristics, recurrence of ADR upon reexposure, or withdrawal of offending drug.

	**Was the allergy or ADR recorded in the patient folder?**					
	**n**	**Yes**	**No**	**NA**	**V**	**p**
*Classification of ADR*					0.269	0.001
Type A: dose related	84	35	41	8		
Type B: nondose related	45	10	17	1		
Type F: failure of therapy	1	0	1	0		
*Was the offending drug stopped?*					0.212	0.049
Yes	125	42	57	26		
No	8	6	2	0		

## Data Availability

The datasets generated during and/or analysed during the current study are available from the corresponding author upon reasonable request.

## Nomenclature

ADE: adverse drug event
ADR: adverse drug reaction
CNS: central nervous system
CVS: cardiovascular system
DTC: Drug and Therapeutic Committee
FDA: Food and Drugs Administration
GIT: gastrointestinal tract
ICP: institutional contact person
JDH: Jasikan District Hospital
LMIC: low- and middle-income countries (LMIC)
pADR: Preventable ADRs
PDHC: Peki Dzake Health Centre
UMC: Uppsala Monitoring Centre
WHO: World Health Organization

## Supporting Information

Supporting Information Additional supporting information can be found online in the Supporting Information section. Supporting Information S1: proposed design and content of allergy card. Supporting Information S2: data extraction tool. Supporting Information S3: select a number that describes ADR observed and responded to Question 9 on the data extraction tool. Supporting Information S4: WHO-UMC causality categories. Supporting Information S5: Schumock and Thornton criteria for assessing preventability of adverse drug events (ADEs). Supporting Information S6: a snapshot of the patients rechallenged with offending drugs after the initial challenge.
