# Supplementary material for: Analysis of Spontaneously Reported Adverse Drug Events: Towards Developing Systems for Preventability
Source: Biomed Res Int. 2024 Aug 30;2024:1906797. doi: 10.1155/2024/1906797 (PMC11379512; doi:10.1155/2024/1906797)
Supplement: Supporting Information — Additional supporting information can be found online in the Supporting Information section. Supporting Information S1: proposed design and content of allergy card. Supporting Information S2: data extraction tool. Supporting Information S3: select a number that describes ADR observed and responded to Question 9 on the data extraction tool. Supporting Information S4: WHO-UMC causality categories. Supporting Information S5: Schumock and Thornton criteria for assessing preventability of adverse drug events (ADEs). Supporting Information S6: a snapshot of the patients rechallenged with offending drugs after the initial challenge. [file 1906797.f1.docx]

**Supplementary material**

**S1:** Proposed design and content of allergy card.

**S2:** Data Extraction Tool.

**S3:** Select a number that describes ADR observed and responded to question 9 on the data extraction tool.

**S4**: WHO-UMC Causality Categories

**S5:** Schumock and Thornton criteria for assessing preventability of adverse drug events (ADEs)

**S6**: A snapshot of the patients rechallenged with offending drugs after the initial challenge

**Front view of proposed Allergy Card**


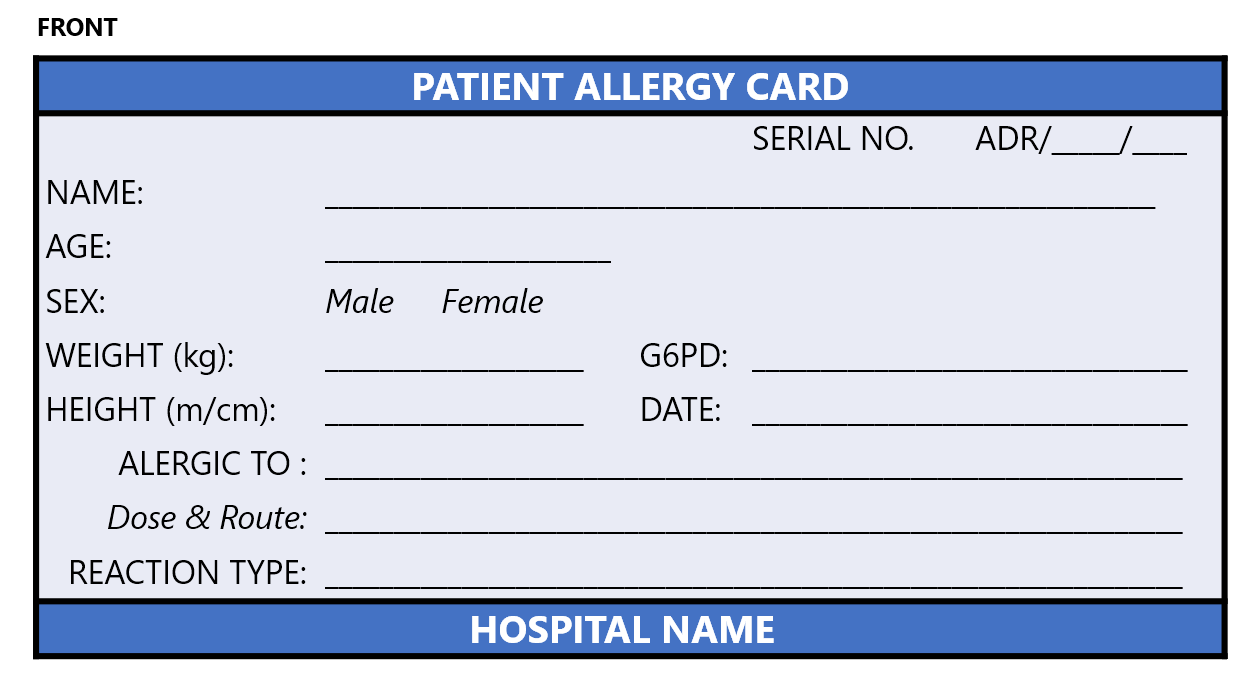


**Back view of proposed Allergy Card**


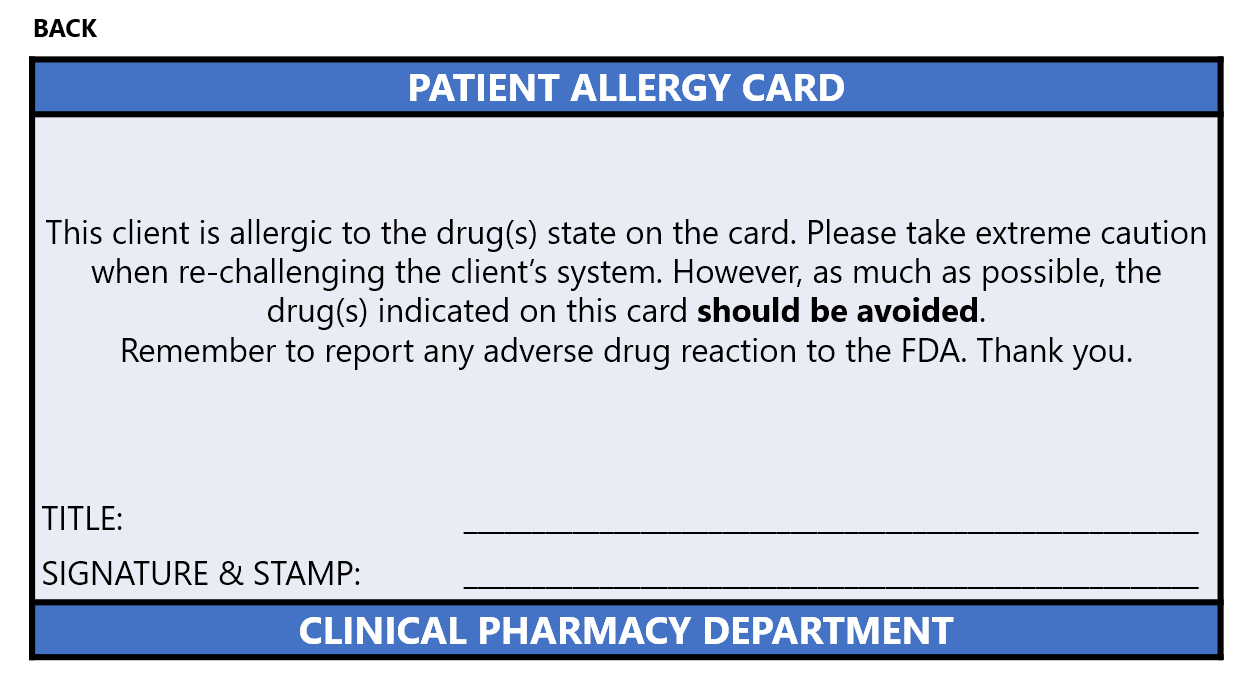


**S2:** Data Extraction Tool

|  | **S3**: Select a number that describes ADR observed and responded to question 9 on the data extraction tool. | |
| --- | --- | --- |
| 1 | Skin Rash |  |
| 2 | Urticaria with wheals |  |
| 3 | Headache (throbbing) |  |
| 4 | Orbital oedema and/or Periorbital pain | |
| 5 | Maculo Papular Rash |  |
| 6 | Dyspnoea |  |
| 7 | Pruritus (Site of Injection) |  |
| 8 | Pruritus (Body)-Generalized body itching | |
| 9 | Swollen lib, face, mouth, tongue, buccal cavity - Angioedema | |
| 10 | Cough |  |
| 11 | Sore throat |  |
| 12 | Anaphylaxis |  |
| 13 | Psychosis |  |
| 14 | Palpitation with chest heaviness |  |
| 15 | Diaphoresis |  |
| 16 | Paresthesia - Unable to move limbs |  |
| 17 | Erythema Multiforme (Generalized skin eruptions) | |
| 18 | Conjunctivitis |  |
| 19 | Oral Mucosal Ulcerations |  |
| 20 | Abdominal Discomfort |  |
| 21 | Other |  |
|  | N/A : Not Applicable |  |

**S4**: WHO-UMC Causality Categories

| **Causality term** | **Assessment criteria*** |
| --- | --- |
| **Certain** | • Event or laboratory test abnormality with a plausible time relationship to drug intake |
|  | • Cannot be explained by disease or other drugs |
|  | • Response to withdrawal plausible (pharmacologically, pathologically) |
|  | • Event definitive pharmacologically or phenomenologically (i.e. an objective and specific medical disorder or a recognised pharmacological phenomenon) |
|  | • Rechallenge satisfactory, if necessary |
| **Probable / Likely** | • Event or laboratory test abnormality, with a reasonable time relationship to drug intake |
|  | • Unlikely to be attributed to disease or other drugs |
|  | • Response to withdrawal clinically reasonable |
|  | • Rechallenge not required |
| **Possible** | • Event or laboratory test abnormality, with reasonable time relationship to drug intake |
|  | • Could also be explained by disease or other drugs |
|  | • Information on drug withdrawal may be lacking or unclear |
| **Unlikely** | • Event or laboratory test abnormality, with a time to drug intake that makes a relationship improbable (but not impossible) |
|  | • Disease or other drugs provide plausible explanations |
| **Conditional / Unclassified** | • Event or laboratory test abnormality |
|  | • More data for proper assessment needed, or |
|  | • Additional data under examination |
| **Unassessable / Unclassifiable** | • Report suggesting an adverse reaction |
|  | • Cannot be judged because information is insufficient or contradictory |
|  | • Data cannot be supplemented or verified |
|  | * All points should be reasonably complied with |

| **S5:** Schumock and Thornton criteria for assessing preventability of adverse drug events (ADEs) | | |
| --- | --- | --- |
| **Section** | **Yes** | **No** |
| **Section A: Definitely preventable ADEs** | | |
| Was there a history of allergy or previous reaction to the drug? |  |  |
| Was the drug involved inappropriate for the patient’s clinical condition? |  |  |
| Was the dose, route, or frequency of administration inappropriate for patient’s age, weight or disease state? |  |  |
| Was toxic serum drug concentration or lab monitoring test documented? |  |  |
| Was there a known treatment for ADEs? |  |  |
| ***Total*** |  |  |
| **Section B: Probably preventable ADEs** | | |
| Was therapeutic drug monitoring or other necessary lab test not performed? |  |  |
| Was the drug interaction involved in ADEs? |  |  |
| Was poor compliance involved in ADE? |  |  |
| Were preventative measures not prescribed or administered to the patient? |  |  |
| ***Total*** |  |  |

**S6**: A snapshot of the patients rechallenged with offending drugs after the initial challenge

| **Folder** | **No. of Repeated ADRs** | **ADR** |
| --- | --- | --- |
| 27792 | 2 | Angioedema |
| 8173/10 | 2 | Angioedema |
| 11235 | 2 | Anaemia, Weakness |
